# A cross-sectional survey of pharmacists to understand their personal preference of brand and generic over-the-counter medications used to treat common health conditions

**DOI:** 10.1186/s40545-016-0066-6

**Published:** 2016-04-21

**Authors:** Mira Patel, Marion Slack, Janet Cooley, Sandipan Bhattacharjee

**Affiliations:** College of Pharmacy, The University of Arizona, Tucson, Arizona USA

**Keywords:** OTC medications, Generic medications, Pharmacists, Survey, Preference

## Abstract

**Background:**

Consumers are hesitant in choosing generic medications as they are under the assumption that they are not as safe nor effective as brand medications. However, pharmacists do have the education and training to know that this is not the case. The aim of this study was to determine pharmacists’ preference of generic versus brand over-the-counter (OTC) medication for their personal use as self-treatment for various health symptoms.

**Methods:**

A prospective, cross sectional study was conducted on 553 licensed pharmacists who were presumed to have expertise in the use of generic and brand name OTC medications. In a single Southwestern state in the United States, from December 2014 to January 2015, a web-based questionnaire was sent to pharmacists to explore their preference of brand and generic medications based on various health symptoms. Thirty-one brand-generic medication pairs were used to identify which medication type pharmacists preferred when asked about nine health symptoms. Frequency counts of pharmacists’ preference of a brand medication or a generic OTC medication overall and for each of the nine health symptoms were determined. Chi-squared analyses and one-way ANOVA were conducted to determine if there were any differences between the preferences of brand and generic OTC medications across each symptom.

**Results:**

The study overall showed that pharmacists preferred generic OTC medications to brand OTC medications (62 to 5 %, respectively). Based on an 11-point rating scale, pharmacists were likely to take OTC generic medications (as their choice of self-treatment) when presented with health symptoms (mean = 7.32 ± 2.88). In addition, pharmacists chose generic OTC medications over brand medications regardless of health symptoms (*p* < 0.001).

**Conclusion:**

Pharmacists who have expertise in medications were shown to prefer using generic OTC medications rather than brand name OTC medications for self-treating a variety of health symptoms. These study findings support the theory that expertise affects preference for generic versus brand name OTC medications. This information can be used to provide consumers the evidence needed to make well-informed choices when choosing between brand and generic medications.

## Background

Of the many ways prescription and over-the-counter (OTC) medications are classified, the two most common categories are by brand and generic. In the United States (US), the development and promotion of more generic medications was made possible through the Hatch-Waxman Act in 1984 [1]. Prior to this Act, the process to develop any medication required a tedious process, thus very few generic medications were produced by pharmaceutical companies. The development of generic medications has allowed consumers in the US to save a substantial amount of money in the range of $8 to $10 billion a year at retail pharmacies and an even greater amount at hospitals [2]. While generic medications are considered to be cost-effective compared to their brand name counterpart, the use of generic medications by consumers is still lacking within the US healthcare system [3]. Many reasons come into play when consumers consider the use of generic medications over brand medications. The primary hesitation is the idea that generic drugs are not as safe or effective as their brand name counterpart. Other research has indicated that even though patients may find generic drugs to be of good value and cost, the majority just prefer not to use them [3]. In addition, patients believe that medications that have been on the market longer are considered safer than newer ones. Also, healthier patients are more concerned about the efficacy of generic medications, similar to that of older adults, who prefer brand name medication to generic medication [3].

While earlier studies have shown consumers’ preference [4, 5] and pharmacists [6, 7] and physicians’ opinion [8] of generic and brand medication, only one study has examined pharmacists’ actual behavior related to their personal use of brand name versus generic drugs for self-treatment. Using data from the 2004–2011 Nielsen Homescan panel, a study was conducted that explored pharmacists purchasing behavior for brand-name or generic OTC medications for headaches [9]. In general, there are times where one population type may be more knowledgeable (i.e., has more information) than another population type, thus leading to potentially different decisions/choices for each group due to the knowledge gap. This economics-based theory is known as information asymmetry. This study was based on that theoretical premise where consumer preference for brand name OTC medications reflects misinformation derived from advertising by companies marketing brand name products. If consumers were fully informed (i.e., had appropriate knowledge and background as is assumed in information asymmetry [10]), they would purchase less costly generic OTC medications. The hypothesis was tested by assuming that expertise, in this case, expertise in medications, could serve as a proxy for perfect information and that by observing the purchasing behavior of pharmacists, the researchers could determine if consumers with expertise in medications behave differently than lay consumers. The study showed that 91 % of pharmacists purchased generic OTC medications for headaches while only 74 % of consumers purchased generics [9]. The authors concluded that consumers could reduce expenditures by $44 billion if they used store brands whenever possible [9]. However, the study was limited as it did not include OTC medications for other symptoms and the sample size was small [9].

In addition to being experts in the area of OTC medications, pharmacists offer other advantages when studying the preference for brand name or generic OTC medications. Pharmacists provide a homogeneous population that is well educated and with a relatively high income. Hence pharmacists’ decisions should be influenced less by differences in education or income. Also, pharmacists can be assumed to have the ability to understand the items on a questionnaire and respond appropriately. Ability to respond accurately to questionnaires and to understand technical language was the rationale for recruiting nurses to the Nurses’ Health Study, a very large longitudinal study of 238,000 nurses on the relationship between nutrition and health [11].

The objective of this study was to identify pharmacists’ preference on their personal use of brand versus generic OTC medications used to self-treat common health conditions/symptoms. Findings from this study would help in extrapolating the results into useful evidence for when pharmacists are advising patients on the use of OTC products as treatments.

## Methods

### Design

A cross-sectional study was conducted by administering an online questionnaire to pharmacists registered in a single Southwestern state in the US. Qualtrics®, an online survey generator, was used to develop and distribute the questionnaire. Although on-line questionnaires typically have a very low response rate, the authors considered this type of data collection as the optimal approach to achieve the purpose of this study, [12, 13] which was to explore the effect of expertise on the use of generic versus brand name OTC medications. The study followed the Declaration of Helsinki in the consideration of ethics in the research study on patients. Thus, a protocol was developed and approved by the University of Arizona Institutional Review Board. Informed consent was provided by participants prior to starting the questionnaire. The questionnaire was made anonymous and no identifiable data was collected. The questionnaire was available for eight weeks, from December 2014 to January 2015, with two follow-up reminders at the 4-week and 6-week points.

### Participants

To be eligible for this study, respondents had to be registered as licensed pharmacists in a single Southwestern state with a valid email address listed with the State Board of Pharmacy. Pharmacy students, pharmacy interns, and pharmacy technicians were not included in the study. The State Board of Pharmacy had 7,265 registered pharmacists with email addresses during the time of the study. Individuals could only participate in the survey if they received a unique link via email to access the online questionnaire.

### Instrument

**Fig. 1:**
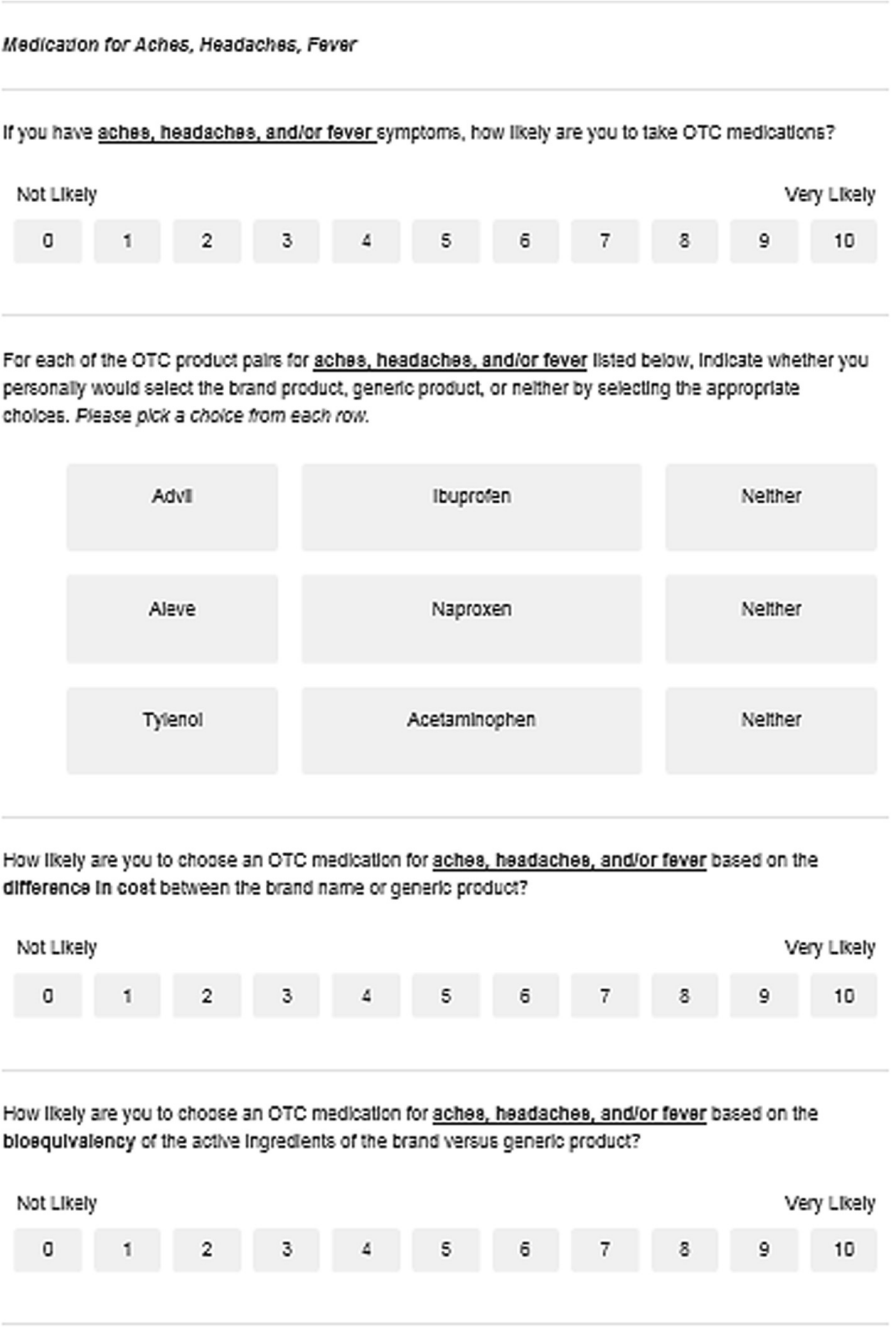
Example of survey questions asked to pharmacists for their personal treatment of aches, headaches, and fever

An original survey was developed for this study; therefore, the majority of questions were not derived from any previously established questionnaires. Throughout the development of the questionnaire, individuals who had a Doctor of Pharmacy degree and had knowledge about brand medications and their generic counterpart were used to make sure the information for each question was valid. The questionnaire contained nine health symptoms (aches, allergies, cough, acid reflux, constipation, insomnia, daytime cold and flu, nighttime cold and flu and pain) with 31 brand-generic medication pairs to explore pharmacists’ preference of brand and generic medications. In addition, a “neither” option was given for each pair if pharmacists did not prefer either type of medication. An example of the type of questions that were asked can be seen in Fig. 1.

The health symptoms and their associated mediations were derived from the 2012 American Pharmacists Association (APhA) OTC Medication Survey [14], which asked pharmacists how many times per week they recommended various categories of OTC medications. From their data, common health symptoms based on their medication categories were chosen. Medications that were recommended at 10 % and greater for each of the nine health symptoms were used in this study’s questionnaire so that less common medication pairs were excluded and would allow for a more concise survey. Topical creams, infant medications, medical equipment, and various other uncommonly used medications were excluded from the survey. Since the APhA study only provided brand name medications, Medscape®, an online resource used by health professionals for medical information, was used to identify the generic counterparts.

In addition, the questionnaire included items that identified cost and bioequivalency influences, health status and general demographics. However, for this study, the main focus was on pharmacists’ preference of brand and generic medications based on the responses they made on the questionnaire.

### Data collection and analysis

The questionnaire consisted of the following sections: (i) general demographics, (ii) preference of brand versus generic OTC medications based on symptoms, (iii) influence of cost and bioequivalency on medication choice, and (iv) health status. The questionnaire was pilot tested with seven graduate students who had a professional pharmacy degree, but were not registered as licensed pharmacists in the state of Arizona. This was done to check for correct formatting, grammar, and content, and to make sure that the data was being collected appropriately. The survey took about 10–15 min for participants to complete, but were allowed as much time to complete as needed and had the ability to stop and restart the survey at a later time. In addition, prior to distributing the questionnaire, settings were established within Qualtrics® to make sure the questionnaire was anonymous with no identifiers (e.g., name, location, IP address) and that participants could not respond to the questionnaire more than once. Qualtrics® was also used to collect the responses from the questionnaire. The investigators formatted and coded the data to be analyzed using Stata®, version 13. Chi-squared tests were conducted to determine if there was a significant difference between the preference of the type of OTC medication based on general demographic characteristics, health symptoms, and health status. One-way ANOVA was conducted to determine the differences in the likelihood of taking OTC medications based on the health symptoms. Multiple logistic regressions were conducted to determine the association of covariates with the preference of brand and generic medications (demographic and work-related variables).

## Results

A total of 553 participants responded prior to the survey being closed (7.6 % response rate). Throughout the survey period, 148 individuals chose to opt out from responding to the survey. The demographic and work-related characteristics of the study participants are presented in Table 1. The typical respondent was a 48-year-old white, male, Doctor of Pharmacy degree holder with a household income of greater than $150,000 who worked full-time in a chain pharmacy as a Staff Pharmacist and had 21 or more years of experience.

**Table 1:** Demographic and work-related characteristics of pharmacist participants surveyed about preference of OTC medications (*n* = 553)

Characteristics	Mean (SD)
Age	48.46 (13.26)
Characteristics	Frequency (Percent)
Gender	
Male	315 (56.96)
Female	238 (43.04)
Race	
Hispanic or Latino Origin	22 (3.98)
White	481 (86.98)
Black or African American	11 (1.99)
Native American or American Indian	2 (0.36)
Asian/Pacific Islander	43 (7.78)
Other	16 (2.89)
Household Income	
Less than $99,999	62 (11.21)
$100,000–$129,000	129 (23.33)
$130,000–$150,000	102 (18.44)
More than $150,000	260 (47.02)
Primary Site of Practice	
Independent Pharmacy	45 (8.14)
Chain Pharmacy	213 (38.52)
Hospital Pharmacy	107 (19.35)
Ambulatory Care Pharmacy	19 (3.44)
Managed Care Pharmacy	37 (6.69)
Long-Term Care Pharmacy	13 (2.35)
Other	119 (21.52)
Job Title	
Staff Pharmacist	239 (43.22)
Clinical Pharmacist	104 (18.81)
Pharmacy Manager	125 (22.60)
Assistant Pharmacy Manager	8 (1.45)
Other	77 (13.92)
Pharmacy Professional Degree	
Doctor Pharmacy	311 (56.24)
Bachelors of Pharmacy	242 (43.76)
Work Status	
Part-Time	96 (17.36)
Full-Time	423 (76.49)
Not Employed/Retired	34 (6.15)
Years of Experience	
Less than 1 Year	1 (0.18)
1–5 Years	89 (16.09)
6–10 Years	73 (13.20)
11–15 Years	62 (11.21)
16–20 Years	44 (7.96)
21+ Years	284 (51.36)

Abbreviations used: *SD* standard deviation

Table 2 illustrates pharmacists’ preference of brand, generic, or neither type of medications was stratified by nine health symptoms that correlated with the medications used as treatment for the health symptoms. There was a statistically significant difference between the three groups based on health symptoms (*p* < 0.001). Additional analyses were performed on brand versus generic, brand versus neither, and generic versus neither. The results indicated that there were statistically significant differences between each of the group pairs based on health symptom. Respondents were least likely to choose brand name insomnia and pain medications (1.4 %), but were more likely to choose brand name cough medications (14.4 %). Also, respondents were less likely to choose generic pain medications (26.3 %), but were more likely to choose generic ache medications (81.2 %). Overall, respondents would prefer not taking brand or generic medications for pain (72.3 %, compared to 1.4 and 26.3 %).

**Table 2:** Pharmacists’ preference of brand, generic, or neither type of OTC medication by type of health symptoms

	Brand	Generic	Neither	Total	*p*-value*
Aches	61 (4.02 %)	1233 (81.2 %)	225 (14.8 %)	1519	<0.001
Allergies	59 (2.4 %)	1941 (77.6 %)	503 (20.1 %)	2503	<0.001
Acid Reflux	173 (5.8 %)	1875 (62.4 %)	957 (31.9 %)	3005	<0.001
Constipation	163 (7.1 %)	1273 (55.6 %)	852 (37.2 %)	2288	<0.001
Cough	148 (14.4 %)	687 (66.6 %)	196 (19.0 %)	1031	<0.001
Pain	29 (1.4 %)	544 (26.3 %)	921 (72.3 %)	1494	<0.001
Insomnia	14 (1.4 %)	561 (55.1 %)	444 (43.6 %)	1019	<0.001
Nighttime Cold and Flu	52 (5.3 %)	536 (54.3 %)	399 (40.4 %)	987	<0.001
Daytime Cold and Flu	41 (4.1 %)	496 (49.7 %)	461 (46.2 %)	998	<0.001
Total	740 (5.0 %)	9146 (61.6 %)	4958 (33.4 %)	14844^a^	

*Chi-squared test for all comparisons

^a^Total responses for all 31 product pairs across 9 symptoms

In Table 3, the means of the likelihood of taking an OTC medication for each of the nine health symptoms are displayed. The results indicated that the health symptoms elicited statistically significant differences in the mean likelihood of taking OTC medications (*p* < 0.05). In addition, based on the mean responses, pharmacists were most likely to take OTC medications for aches, while least likely to take medications for insomnia. While respondents were likely to take OTC medications for pain based on the mean responses (8.01), there was a contradiction in pharmacists’ overall preference between brand, generic, and neither choices in that pharmacists did not prefer any of the pain medications for the product pairs that were provided in this survey or chose not to self-treat this specific symptom.

**Table 3:** Pharmacists’ responses for the likelihood of taking OTC medications by health symptoms

Health symptom	Mean response^a^ (SD)
Ache	8.60 (2.02)
Allergies	8.36 (2.29)
Pain	8.01 (2.54)
Nighttime Cold and Flu	7.60 (2.92)
Acid Reflux	7.42 (2.97)
Daytime Cold and Flu	7.37 (2.95)
Cough	7.15 (3.01)
Constipation	6.52 (3.46)
Insomnia	4.84 (3.77)
Average of Mean Responses	7.32 (2.88)

Abbreviations used: *SD* standard deviation

^a^From an 11-point Likert Scale, where 0 = Not Likely and 10 = Very Likely

The multivariate logistic regression analysis revealed a statistically significant association between races, work statuses, years of experiences with the use of generic OTC medications for the treatment of pain. No other significant relationships were identified between the use of generic OTC medications and other demographic and other work related variables measured in this study.

## Discussion

The most important finding of this study is that pharmacists had an overwhelming preference for generic OTC medications compared to brand OTC medications (62 to 5 %) for symptoms that they personally experience. Furthermore, there were pharmacists that preferred to not use the listed (brand or generic) OTC medication, but rather may have preferred another product entirely or to not treat the symptom. The preference for generic medication was shown overall and across all health symptom categories. For aches, allergies, cough, acid reflux, constipation, insomnia, daytime cold and flu, nighttime cold and flu, and pain, pharmacists chose generic medications over brand medications. Also, when presented with a health symptom, pharmacists indicated that they were quite likely (overall mean rating of 7.3 on a scale where 10 = very likely) to take an OTC medication for almost all symptoms, except for insomnia (mean rating = 4.8).

An earlier study on pharmacists’ was done to identify pharmacists’ choice of store-brand (generic) and brand named headache and pain medication. Their results corresponded with this study’s findings in that pharmacists are more informed about the active ingredients in medications, thus were more likely to choose store-brand or generic medications than other consumers [9]. Both studies demonstrated information asymmetry where the pharmacists were more knowledgeable in the area of pharmaceuticals compared to other consumers, thus were able to make a well-informed decision.

The overwhelming preference for generic OTC medications found in this study are consistent with the findings of *Bronnenberg* et al. that 91 % of pharmacists in their sample purchased generic OTC headache medications [9]. A study of Irish pharmacists’ perceptions and attitudes revealed that 98 % of pharmacists believed that the quality of generics was similar to brand-name medications, however, 7 % stated that they would prefer the original product for their personal use [15]. The 7 % of Irish pharmacists is similar to the 5 % of pharmacists in this sample who prefer brand name medications. However, the findings of this study may not be generalizable to all other countries. A study in New Zealand assessed pharmacists’ perceptions of generic medications in general; the findings indicate that the perceptions of generic medications were less positive than in the United States; 65 % of New Zealand pharmacists believed that brand name medications were of higher quality than generic medications [7].

Pharmacists who had more years of experience were less likely to choose generic medications compared to those who had less years of experience. This supports previous research, which indicated that pharmacists who had never practiced in a pharmacy setting were more likely to understand generic medication than those who had more years of practice [7]. This can be due to the assumption that there is more information and education given to pharmacy students now than in the past. During the 1980s, pharmacists believed that making substitution decisions had great risks so they lacked the confidence in making generic substitutions. In addition, they lacked the confidence in the quality and therapeutic effects compared to brand medications [16]. Thus, this research study shows that individuals who have had ten years of experience or less from this time point were more likely to prefer generic medications compared to those who had eleven years or more of experience. Previous studies indicated that pharmacists were hesitant to suggest generic medications due to risks, especially based on the extent of the medical condition of the patient [17]. However, education has changed throughout the years and laws have been implemented, which has required pharmacists to dispense generics appropriately and has allowed them to gain more knowledge about generic medications to feel comfortable in substituting them for brand medications [18].

Also, from the results, it can be seen that pharmacists prefer generic medications (*n* = 9146) versus brand medications (*n* = 740) or neither types of medication (*n* = 4958). This was based on the counts of each pair of medications for the nine different health symptoms. In almost all symptoms, except insomnia, the preference of generic medication was far greater than brand medication, while in insomnia; pharmacists preferred the option of neither brand nor generic medication. This may have been due to the fact that insomnia is not as common of a symptom compared to the other health symptoms that were used in this study. Pharmacists’ preference of generic medications can be attributed to their knowledge in the area of pharmacy. They are knowledgeable from the degrees they have attained, doctor of pharmacy (*n* = 311) and bachelors of pharmacy (*n* = 242), thus they are able to make an informed choice on what medications they want to take themselves.

Because pharmacists have relatively high incomes, it can be assumed that pharmacists were not selecting generic OTC medications because they cannot afford brand name OTC medications. However, use of generic OTC medications can result in significant savings to the consumer. Bronnenberg et al., 2013 estimated that total expenditures for headache remedies would decrease by 13 % if consumers were as knowledgeable as pharmacists [9]. This further supports that pharmacists have confidence in generic medications and will choose them, regardless of the price and their ability to afford both types of medications.

It is not clear what the implications of pharmacists’ overwhelming preference for generic OTC medications are for the consumer. Several studies have shown that how health care professionals care for themselves is not necessarily related to how they care for patients. In a study of pharmacists’ use of skin cancer prevention strategies, personal use of strategies to prevent sunburn were not correlated to pharmacists’ counseling of patients about prevention strategies [19]. Incongruence between personal behaviors and patient related behaviors suggests that factors other than personal practice (e.g., expectation of counseling) have a strong influence on professional behaviors. Several studies have shown that physicians treat themselves differently than they treat patients. One study examined the rate of C-sections in women physicians and found that women physicians were 10 % less likely to have a C-section than other women. They attributed the difference to women physicians’ expertise in medical care [20]. In a second study of the health-related behaviors of women physicians, the authors found that women physicians’ health-related behaviors exceeded the goals of Healthy People 2000 [21].

The influence of the study findings on consumer behavior is not apparent at this time point. At least one study has shown that informing consumers that the active ingredients are similar does not affect their choice of generic medications [22]. Another study showed that women who had Medicaid insurance believed that generics were less expensive (98 %) and a better value (82 %) but only 45 % preferred to take generics themselves [23]. Perhaps for the lay consumer, a non-scientific argument would have more effect. For example, rather than presenting scientific information, discuss how pharmacists themselves or their family members prefer to take generic OTC medications.

### Limitations

This study has several limitations and hence interpretations should be drawn with caution keeping the following limitations in mind. First, the sample was of pharmacists that were licensed in a single Southwestern state, therefore the study results do not report the prevalence of use of generic OTC medications versus brand name OTC medications for all pharmacists. Also, due to time constraints, the online survey was only available for two months, thus if more time was allotted, more participants may have provided responses. In addition, the listed medications for each health symptom were not comprehensive of all possible medications, but rather those that were commonly used. The use of an “Other” text box may have been beneficial in this case to attain all possible responses from the pharmacists. Lastly, the questions in the survey asked participants about their preference of taking brand or generic OTC medications versus their actual behavior.

## Conclusion

The findings of this study indicated that pharmacists had a significant preference for choosing generic medications versus brand medications across the health symptoms of aches, allergies, cough, acid reflux, constipation, insomnia, daytime cold and flu, nighttime cold and flu and pain. Overall, the study provided evidence that pharmacists (experts) prefer generic medication over brand medication for their personal use as self-treatment, supporting the theory that experts have the information to make well-informed decisions. Future research can be conducted on the families of pharmacists to see what the pharmacists recommend to the family members as treatment options. In addition, research can be done on pharmacists’ choice of brand and generic OTC medications for use when treating their own children. Also, other health symptoms and medication types (e.g., topical treatment) besides the ones used in this study can be further researched.
